# Survey of human papillomavirus types and their vertical transmission in pregnant women

**DOI:** 10.1186/1471-2334-13-109

**Published:** 2013-02-27

**Authors:** Ying Hong, Shu-Qin Li, Ya-Li Hu, Zhi-Qun Wang

**Affiliations:** 1 Nanjing Drum Tower Hospital, The Affiliated Hospital of Nanjing University Medical School, 321 Zhongshan Road, Nanjing 210008, China; 2 Nanjing Medical University, Nanjing 210008, China; 3 Nanjing University of Chinese Medicine, 210008, Nanjing, China

**Keywords:** Human papillomavirus, Pregnancy, Vertical transmission

## Abstract

**Background:**

The prevalence, genotypes, and vertical transmission characteristics of human papillomavirus (HPV) among pregnant women from Nanjing, China was investigated.

**Methods:**

Cervical cells were collected from healthy pregnant women (n = 3139; stage of gestation, 24.6 ± 2.1 weeks) for cytological evaluation and determination of HPV infection status. Exfoliated oral and genital cells were collected from neonates (<1-day-old, n = 233) whose mothers were positive for HPV DNA. We used HPV Gene Chip technology with 23 HPV genotype probes to conduct our analysis.

**Results:**

Overall prevalence of HPV DNA among pregnant women was 13.4% (422/3139). The most frequently detected HPV genotypes were HPV-16 (29.6%, 125/422), -18 (14.7%, 62/422), and -58 (14.2%, 60/422). The rate of concordance for HPV DNA in maternal-neonatal pairs was 23.6% (55/233), with HPV type-specific concordance occurring in 26 cases. A higher prevalence of HPV DNA was apparent in female neonates compared with males (17.7 *vs*. 11.6%).

**Conclusions:**

The prevalence of cervical HPV DNA in pregnant women from Nanjing was low, with vertical transmission rates slightly higher. From our findings, we concluded that there was efficient vertical transmission of three HPV genotypes, with HPV-16 the most prevalent type in pregnant women and newborn babies.

## Background

Globally, cervical cancer is the second-most commonly occurring cancer in women. In recent years, the occurrence of sexually transmitted infections (STIs) involving human papillomavirus (HPV) has been increasing in China, and are now second only to the incidence of STIs for gonorrhea [1]. HPV has been recognized as the primary cause of cervical cancer, papillomatosis, and anogenital warts [2,3]. Low-risk, non-oncogenic forms of HPV are associated with anogenital warts and laryngeal papillomatosis; high-risk, oncogenic types are associated with cancers of the cervix, anogenital areas, head and neck [4].

In the early 1950s, a study revealed that pregnant women infected with HPV were able to vertically transmit the virus to neonates, resulting in infantile anal and genital condyloma acuminatum, congenital conjunctival papilloma, and juvenile laryngeal papillomatosis [5,6]. A recent report involving 88 cases where the sexual life history of females was followed indicated that congenital infection of HPV might result in an increased risk of genital condyloma acuminatum [7]. However, geographic regions display different HPV genotype distribution among ethnicities. The current methods of virus detection are based upon rates of congenital HPV infection, which have been reported at 0–80.9% [8].

The five most prevalent HPV genotypes associated with genital and oral cancers in females are HPV-16, -18, -31, -58, and -52 [9]. Previous surveys in China have found HPV-52 and -58 to be more prevalent than HPV-16 in some areas [10,11]; this distribution is consistent with those found in Japan and eastern Africa [9]. Most studies have found a higher prevalence of HPV DNA among pregnant women compared with those who are not pregnant [12-14].

It was previously shown, using DNA hybridization techniques, that HPV could not be detected in 11 oropharyngeal samples taken from neonates (1-day-old). However, from an age of 5 weeks up to 18 months, HPV DNA could be detected [15]. This suggests that the virus may be carried by the neonate even though it could not be detected by DNA hybridization. Mant *et al*. [16] reported using a more sensitive polymerase chain reaction (PCR) detection method to detect HPV-16 DNA in 20 neonatal oropharyngeal samples. After 30 months, eight samples were positive for virus.

Although there is overwhelming evidence for the sexual transmission of high-risk HPV genotypes, several studies have examined other routes, such as vertical transmission before or during childbirth, direct contact during labor, or horizontal transmission among children through contact with infected skin lesions [17,18]. However, similar studies of transmission modes in different regions have obtained disparate results. Studies of vertical HPV transmission have reported a wide range of neonatal infection rates. These conflicting findings are primarily because of the differences in populations and experimental techniques, and might have been influenced by factors such as sex, type of delivery, and maternal status before delivery.

It is important to examine the distribution of HPV genotypes found in pregnant women, as it can guide the development of genotype vaccines for the prevention of cancer due to HPV infection [19]. The prevalence of vertical HPV transmission may have an important impact on clinical handling and vaccination strategies for infected women during pregnancy. We sought to determine the prevalence, types, and vertical transmission characteristics of HPV in pregnant women from Nanjing (Jiangsu Province, China).

## Methods

### Participants

Healthy pregnant women (n = 3139) were recruited, from a sample population of 11,696 women, who had their first obstetric examinations at the Affiliated Drum Tower Hospital (Nanjing University Medical School, China) between January 2006 and April 2010. A follow-up study was conducted after delivery. The exclusion criteria for this study were: (1) near miscarriage or abnormal vaginal bleeding; (2) cervical lesions apparent upon simple visual inspection during gynecological examination; (3) sexual intercourse and/or vaginal medication in the previous 3 days; (4) HPV detected by cervical cytological examination within 1 year; (5) mental or physical incompetence; (6) *in vitro* fertilization (IVF); and (7) refusal of gynecological examination. There were 1088 pregnant women that were excluded due to the exclusion criteria. The mean age of the 3139 participants was 29.9 years (range, 20–44 years). Most (81.5%, 2558/3139) women were in the 22^nd^–26^th^ week of gestation upon enrollment in the study. All participating women provided written informed consent.

### Data collection

Sociodemographic information for all participants, from January 2006 until April 2010, was obtained, along with gestational age when samples were collected, and parity data. A follow-up study was performed after delivery. Samples were collected from all pregnant women and from 233 infants of the 422 HPV-positive women due to some mother refusing examination and sampling of the infant. The sample collection was carefully executed to prevent cross-contamination between subjects and anatomical sites by using disposable equipment and changing the bed lining and collector’s gloves between each subject. Sample collection was carefully performed to prevent cross-contamination between subjects and anatomical sites, by using disposable equipment, and changing bed linen and collector’s gloves before sampling each subject. During gynecological examination, two cervical smears were collected from each participant for cervical cytological analysis and HPV detection. A cervix brush was used to obtain exfoliated cells from the squamocolumnar junction of the cervix. The collection instrument was then rinsed in transport medium [20,21]. A collection device (Decipher Bioscience, Shenzhen, China) was used to obtain cervical exfoliated cells for HPV-DNA detection. In accordance with the manufacturer’s instructions, samples were collected by scraping the uterine cervical canal with a cervical brush and sent to a diagnostic laboratory within 24 h. Less than 24 h after birth, exfoliated oral and genital cells were collected from the subset of clean neonates using the same collection device used for HPV DNA detection. Close contact of the child with the mother was avoided to prevent contamination with the mother’s exfoliated cells. Oral samples were obtained by allowing the neonate to suck on the sampler for about 10 s. Genital samples were collected from males by gently swabbing the glans penis and the inner mucosal part of the prepuce. Specimens were obtained from females by gently swabbing the vulvar mucosa. Tips containing cellular material were then placed into transport medium tubes and immediately stored at -20°C.

### Laboratory methods

Cytological specimens were sent to a diagnostic pathology laboratory for examination, and prepared using a ThinPrep 2000 Processor (AutoCyte Inc., Burlington, NC, USA), according to the manufacturer’s instructions. Cell suspensions were used to prepare liquid-based cytology slides that were examined using the ThinPrep imager by trained cytologists. The computer-based imaging technology employed for the primary cervical screening for epithelial cell abnormalities used the following classifications [22]: (1) negative for intraepithelial lesion or malignancy (encompassing the previous category of “within normal limits”); (2) atypical squamous or glandular cells of undetermined significance (ASCUS, AGUS); (3) atypical squamous cells, not excluding high-grade squamous intraepithelial lesion (ASC-H); (4) low-grade squamous intraepithelial lesion (LSIL); (5) high-grade squamous intraepithelial lesion (HSIL); and (6) squamous-cell or adenomatous carcinoma (SC, AC). Many studies have confirmed that ThinPrep liquid-based cytology has improved accuracy and reading times, enhancing laboratory productivity and clinical outcomes. This technology also allows HPV testing of the same sample [23,24].

HPV genotyping was conducted using PCR, DNA hybridization, and an HPV genotyping DNA chip (Decipher Bioscience). An HPV typing kit (Guangdong Hybribio Biotech) was used for quality control. An HPV GenoArray Test Kit (HybrMax), based on “Flow-through Hybridization” and certified for use by the State Food and Drug Administration, was also used for the rapid detection of 21 HPV genotypes. DNA was extracted from samples and centrifuged (13,000 rpm for 10 min). PCR assays were performed in reaction tubes containing 5 μL of DNA and 20 μL of reaction buffer containing specific biotin-tagged oligonucleotide primers. Thermal cycling conditions involved preheating at 50°C for 15 min, then an initial denaturation step at 95°C for 10 min, followed by 40 cycles of 30 s at 94°C, 90 s at 42°C, and 30 s at 72°C, and then a final extension step at 72°C for 5 min. Reverse hybridization was performed using a line-probe assay to enable the PCR products hybridized with specific probes to fix to membranes. Chemical colorization [0.1 M sodium citrate, tetramethylbenzidine (TMB) substrate, 30% H_2_O_2_, 30 min] was then conducted to visualize results.

### Statistical analyses

Statistical analyses were performed using the SPSS version 17.0 for Windows (SPSS Inc., Chicago, IL, USA). Logistic regression was used to estimate odds ratios (ORs) and 95% confidence intervals (CIs) to determine factors associated with HPV infection. The influence of delivery type and neonate gender on neonatal HPV infection was analyzed using the χ^2^ or Fisher’s exact test. A *P*-value less than 0.005 was considered statistically significant.

## Results

The risk factors for HPV infection, and the cervical prevalence of one or more HPV genotypes in the 3139 participants is outlined in Table 1. Overall prevalence of HPV DNA was 13.4% (422/3139). The mean age of uninfected women at delivery was 29.9 ± 3.5 years (mean ± standard deviation; range, 20–44 years), while that of HPV-positive women was 27.9 ± 3.5 years (range, 20–40 years). Prevalence of HPV was significantly higher in women aged 24 years or younger (χ^2^ = 12.07, *P* = 0.001). Prevalence of HPV in women experiencing their first pregnancy was 13.8% (234/1692; χ^2^ = 0.67, *P* = 0.41). Parity did not appear to be associated with HPV prevalence (OR for ≥3 gestations *vs*. 1–2 gestations = 1.2, 95% CI: 0.91–1.58). Most women in this study (72.4%) had a level of education that went past secondary school. A significant difference was seen in HPV prevalence among women with different education levels (OR for ≤11 *vs*. ≥18 years = 4.4, 95% CI: 1.8–11.1; OR for 12–17 years *vs*. ≥18 years = 32.0, 95% CI: 1.2–7.4; *P* < 0.01). Abnormal cervical cytological results were found in 42 (1.3%) women, with an HPV prevalence of 76.2% (32/42).

**Table 1:** Risk factors for prevalence of HPV DNA among pregnant women

**Risk factor**	**Number of cases (%, HPV+)**	**Odds ratio**	**95% confidence interval**
***Age (years)***			
≤24	62/348 (17.8%)	1.2	0.85–1.67
25–29	243/2032 (12.%)	0.75	0.58–0.95
≥30	117/759 (15.4%)	Reference	-
***Parity***			
1–2	348/2662 (13.1%)	Reference	-
≥3	73/477 (15.3%)	1.2	0.91–1.58
***Education (years)***			
≤11	150/865 (17.3%)	4.4	1.78–11.09
12–17	267/2163 (12.3%)	3.0	1.21–7.39
≥18	5/111 (4.5%)	Reference	-
***Cytological result***			
Normal	390/3097 (12.6%)	Reference	-
ASCUS	22/32 (68.7%)	15.3	7.18–32.49
LSIL	7/7		
HSIL	3/3		
	422/3139 (14.4%)		

**Figure 1:**
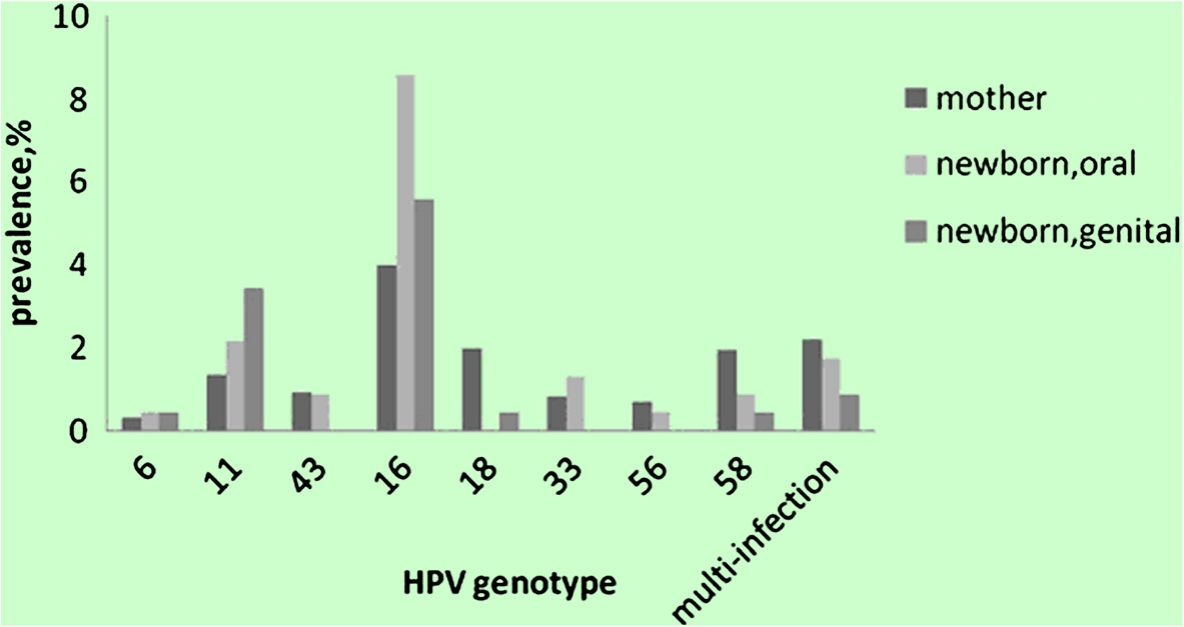
Prevalence of HPV-6, -11, -43, -16, -18, -33, -56, -58 (and multiple infections) among 422 HPV DNA-positive women and 55 HPV DNA-positive newborns.

Figure 1 shows the HPV genotypes and frequencies of detection in mothers and neonates. A high proportion of the HPV-positive women (95.5%, 403/422) were infected with high-risk HPV genotypes. The most frequently detected HPV genotypes were HPV-16 (29.6%, 125/422), -18 (14.7%, 62/422), and -58 (14.2%, 60/422). The HPV-16 and -18 genotypes, which can be vaccinated against, accounted for 44.3% (187/422) of HPV infections. Of the 422 HPV-positive samples, 353 (83.6%) contained a single virus genotype, while 69 (16.3%) were positive for two or more HPV genotypes. HPV-16 DNA was present in 46.4% (32/69) of samples that were identified as containing multiple HPV genotypes.

In 23.6% (55/233) of neonates, the oral and/or genital mucosa were positive for HPV DNA. No significant difference was seen between male and female infants (χ^2^ = 1.64, *P* = 0.268), or between the type of delivery (χ^2^ = 0.283, *P* = 0.71). The HPV-16 genotype accounted for the majority (56.4%, 31/55) of infections in infants. In 233 mother-neonate pairs, the HPV DNA concordance rate between mothers and newborns was 23.6% (55/233). HPV type-specific concordance occurred in 26 cases, with HPV-16 the most frequently detected genotype (18/26), followed by HPV-11 (2/26) and -31 (2/26) (Table 2).

**Table 2:** Neonatal HPV status among 233 infants with HPV-positive mothers

**HPV status**	**Male (cases)**	**Female (cases)**	**Total (cases)**
HPV+	12	23	35
HPV-	91	107	198
Total	103	130	233

Delivery type did not significantly affect the rate of vertical transmission from HPV-positive mothers, where 58.4% (36/233) of the tested neonates were delivered vaginally and 41.6% (97/233) were delivered by cesarean sections (χ^2^ = 0.119, *P* > 0.005). The HPV DNA concordance rate between these 233 mother-neonate pairs was 23.6% (55/233). HPV type-specific concordance occurred in 26 cases (11.2%) and HPV-16 was the most frequently detected genotype (Table 3).

**Table 3:** Neonatal HPV status by delivery type

	**Cesarean section**	**Vaginal delivery**	**Total**
HPV+	16	19	35
HPV-	81	117	198
Total	97	136	233

Among the 233 infants with HPV-positive mothers, 103 (44.2%) were males and 136 (58.4%) were born by vaginal delivery. No significant difference was seen between male and female infants (χ^2^ = 1.64, *P* = 0.268), or between different delivery types (χ^2^ = 0.283, *P* = 0.71).

## Discussion

In this study we examined the vertical transmission of HPV DNA in a large patient population. Our findings revealed that type-specific HPV discordance was high, suggesting other infection routes besides the maternal-neonate route. Although the prevalence of HPV in mothers with abnormal cervical cytology (76.2%) was much higher than that of mothers with normal cytology, none of the 19 neonates from mothers with abnormal cytology were HPV-positive. There was no significant influence of delivery type on the vertical transmission of HPV, which is consistent with reports of congenital condyloma after cesarean section without premature rupture of membranes [25]. Delivery *via* cesarean section did not eliminate the risk of vertical transmission, suggesting that such transmission of HPV DNA may occur before birth by the transplacental route. Several studies have found HPV can be found in the amniotic fluid, fetal membranes, and cord blood [26-28]. Disparity of HPV genotypes in maternal and neonatal HPV DNA-positive samples occurred in 52.7% of cases, indicating transmission by fomites (i.e., contaminated instruments), contact with the child after birth, or experimental contamination of the samples might have occurred.

Ping *et al*. [1] previously reported the detection of HPV-6, -11, -16, and -18 in cervical and vaginal secretions, and in peripheral venous blood samples in women at the latter stages of gestation. Following birth, neonate pharyngeal secretions were also positive for HPV-6, -11, -16, and -18. The prevalence of HPV DNA in the cervical, vaginal exfoliated cells has been shown to be low during the early stages of pregnancy (5/30 cases) and in the second trimester (12/42cases), with prevalence increasing in the final trimester (23/31 cases). In the third trimester, this prevalence was significantly different compared with that during the first two trimesters. Successive examinations on positive women for HPV-6, -11, -16, and -18 in infants at time of birth, 48 ~ 72 h and 6 weeks after birth showed positive HPVDNA in the nasopharyngeal secretion of 13, 6 cases and 1 case with respect to the examining periods. The positive cases were mainly infected by HPV- 16, 18.

Pregnant women in Jiangsu Province usually begin routine obstetric care around the 24^th^ week of gestation; 81.5% of the women in our study were at weeks 22–26 of gestation. Given this small interval of gestational age, it was not possible to examine the influence of this variable on HPV infection in pregnant women. It was previously found that HPV infection in pregnant women was primarily associated with maternal age and education. The influence of education level on HPV infection is most likely explained by the early age of first sexual intercourse and first pregnancy [28].

The microarray used in this study was developed by Decipher Bioscience to detect 23 HPV genotypes. As a diagnostic tool, the application of microarray technology has the advantage of discriminating HPV genotypes and detecting infection of multiple HPV subtypes [29]. In a population-based cross-sectional screening study of 1137 women aged 15–59 years in Shenzhen, Li *et al*. [30] confirmed the clinical value of this technology for the detection of HPV in cervical cancer screening. Halfo *et al*. [31] found a 93% concordance (k = 0.82) between the HC-II assay and microarray technology, indicating that microarrays are highly sensitive and specific in the detection of HPV genotypes. The PCR conducted to detect HPV DNA in pregnant women and showed that viral DNA could be detected in exfoliated cervical and vaginal cells. Additionally, this assay also detected DNA for HPV-16, -11, -6, -35, -31, -58. In all cases except one, the HPV DNA detected in the newborn was the same genotype as that detected in the mother [32,33].

Immunological or hormonal changes may alter the prevalence of HPV and its clearance during pregnancy [34]. Some researchers have reported decreased clearance of high-risk HPV types in the first two trimesters of pregnancy [34,35], contributing to a high prevalence of HPV during pregnancy. The prevalence of HPV DNA among pregnant women that we observed (13.4%) was similar to that reported by a multicenter epidemiological survey for the general female population of China (13.8%) [36]. Similarly, Zhang *et al*. [37] found no significant difference between prevalence of HPV DNA and the pregnancy status of 711 women in Beijing.

The prophylactic bivalent HPV 16/18 vaccine manufactured by GlaxoSmithKline is currently in phase-III clinical trials in Jiangsu Province. Few studies have evaluated the safety of this vaccine for pregnant women in China. Given that primary target population of the HPV vaccine is women of a reproductive age, the risks associated with its administration during pregnancy are important factors affecting personal decisions and public health policy. In a study of 3599 pregnancies, Wacholder *et al*. [15] detected a small increase in the risk of miscarriage for pregnancies conceived within 3 months of vaccination.

The present study found a high prevalence of HPV among pregnant women in Nanjing, the urban center of a highly developed province in China. This prevalence did not differ significantly from that found among the general female population of China. Although some vertical transmission was documented, the high rate of discordance in the HPV genotypes of mothers and neonates indicates the need for efforts to prevent horizontal transmission. Subsequent investigations should examine the role of viral load in vertical transmission.

This large-scale investigation evaluated the prevalence of HPV infection in pregnant women and the rate of vertical transmission. The most frequently detected HPV genotypes in mothers and neonates were HPV-16 and -18, consistent with the target genotypes of HPV vaccines. Currently, no vaccine recommendations for pregnant women have been provided and it is hoped that the current study provides preliminary information that can guide appropriate vaccination strategies for mothers and newborns.

## Conclusion

The most frequently detected HPV genotype in pregnant women and newborns was HPV-16.

### Ethics approval

This study was approved by the Affiliated Drum Tower Hospital, and Nanjing University Medical School Ethics Committees.

## Abbreviations

HPV: Human papillomavirus

## Competing interests

The authors declare that they have no competing interests.

## Authors’ contributions

YH conceived and designed the project, and analyzed data. S-QL participated in data collection. Y-LH provided guidance on this project. Z-QW participated in part of the data collection. All authors have read and approved the final manuscript.

## Pre-publication history

The pre-publication history for this paper can be accessed here:

http://www.biomedcentral.com/1471-2334/13/109/prepub
